# Dr. Wm. H. Loughhead, Jr.

**Published:** 1912-11

**Authors:** 


Dr. Wm. H. Loughhead, Jr., Buffalo 1891, of Andover, died at Hornell, Sept. 12, following an operation for appendicitis, aged 52.

